# 522 Food Deserts and Burn Wound Healing - Does Geography in an Urban Environment Matter?

**DOI:** 10.1093/jbcr/irac012.153

**Published:** 2022-03-23

**Authors:** Brienne Donovan, Jeffrey Anderson, Chinaemelum Akpunonu, Lisa Rae, Huaqing Zhao

**Affiliations:** Temple University, Philadelphia, Pennsylvania; Temple University, Philadelphia, Pennsylvania; Temple University, Philadelphia, Pennsylvania; Temple University, Philadelphia, Pennsylvania; Temple University, Philadelphia, Pennsylvania

## Abstract

**Introduction:**

Many burn injury victims in the United States live in regions designated as food deserts. The United States Department of Agriculture (USDA) defines food deserts as low-income areas where a substantial number of residents do not have access to a supermarket. Nutrition is known to be critical to wound healing. The purpose of this investigation was to determine if there is a relationship between residence in a USDA designated food desert, burn patient cormorbidities, and wound healing at an urban academic medical center.

**Methods:**

We performed a retrospective review of burn injured patients at an ABA verified urban academic burn center between September 2018 and April 2021. Inclusion criteria were burn injury of less than 20% total body surface area (TBSA), age ≥ 18, and single operation for split thickness skin grafting. Zip codes were used in conjunction with the USDA Food Access Research Atlas to classify residence in food deserts. The primary outcome was donor site time to healing. A multivariable logistical regression analysis was performed to evaluate risk factors for poor wound healing at an urban academic burn center and to determine if residence in a USDA delegated food desert was one of those risk factors.

**Results:**

A total of 150 patients were identified for inclusion from September 2018 through April 2021. There were 73 women (48.7%) and 77 men (51.3%). The median age was 48.5 (IQR 34.0, 58.0). The average body mass index (BMI) was 28.2 (6.6). Age (p=0.60), sex (p=0.35), hypertension (p=0.74), chronic obstructive pulmonary disease (p=0.076), hyperlipidemia (p=0.77), congestive heart failure (p=0.47), and BMI (p=0.37), and time to donor site healing (p=0.55) were not significantly different between patients who lived in food deserts and those who did not. Patients who lived in food deserts, however, had a higher incidence of diabetes (p=0.05). The multivariable model also shows that time to healing is not different between patients who live in food deserts and those who did not. However, the multivariable model shows that patients with diabetes have an increased time to healing (p=0.002).

**Conclusions:**

Residence in a USDA delegated food desert does not significantly influence time to healing of donor sites in burn injured patients. However, diabetes is significantly higher in patients who live in USDA delegated food deserts, and diabetes demonstrates a significant delay in wound healing. This is the first study comparing residence in a USDA food desert, burn patient comorbidities, and time to wound healing in an urban burn population.

